# Unusual fast secondary relaxation in metallic glass

**DOI:** 10.1038/ncomms8876

**Published:** 2015-07-24

**Authors:** Q. Wang, S.T. Zhang, Y. Yang, Y.D. Dong, C.T. Liu, J. Lu

**Affiliations:** 1 Laboratory for Microstructures, Institute of Materials Science, Shanghai University, Shanghai 200072i, China; 2 Center for Advanced Structural Materials, Department of Mechanical and Biomedical Engineering, City University of Hong Kong, Kowloon Tong, Kowloon, Hong Kong

## Abstract

The relaxation spectrum of glassy solids has long been used to probe their dynamic structural features and the fundamental deformation mechanisms. Structurally complicated glasses, such as molecular glasses, often exhibit multiple relaxation processes. By comparison, metallic glasses have a simple atomic structure with dense atomic packing, and their relaxation spectra were commonly found to be simpler than those of molecular glasses. Here we show the compelling evidence obtained across a wide range of temperatures and frequencies from a La-based metallic glass, which clearly shows two peaks of secondary relaxations (fast versus slow) in addition to the primary relaxation peak. The discovery of the unusual fast secondary relaxation unveils the complicated relaxation dynamics in metallic glasses and, more importantly, provides us the clues which help decode the structural features serving as the ‘trigger' of inelasticity on mechanical agitations.

## Abstract (web summary)

Mechanical relaxation processes in glasses can provide information on the structural and mechanical properties of glasses. Here, the authors observe a fast secondary relaxation process in La-based metallic glasses, providing information on the inelasticity of metallic glasses.

## Graphical abstract

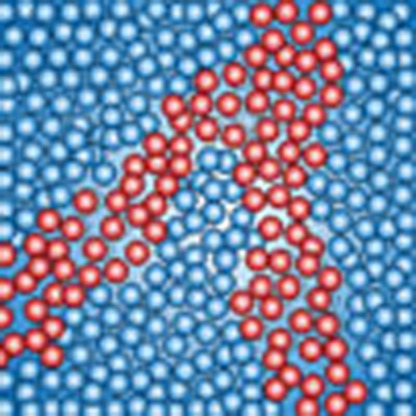

Since the ancient times, glass has been an important engineering material that was used by humans in tools making. Today, there are many kinds of glasses, natural or man-made, which are widely employed in different engineering applications, such as oxide glass for optics, polymeric glass for biotechnology, colloidal glass for medicine, and silicon glass for electronics^1^,^2^,^3^,^4^. Despite the importance of the glassy materials, however, their structure has been remaining a longstanding problem in materials science and condensed matter physics^5^. From the structural viewpoint, glassy materials possess no long-range translational symmetry^6^, hence defying the use of conventional tools for a thorough structural understanding. From the viewpoint of structural dynamic heterogeneity^6^,^7^,^8^,^9^, glassy materials contain regions of different relaxation times, which could give rise to different relaxation spectra on external agitation^10^. Therefore, the relaxation behaviour of glassy materials is very important, which can shed light on the hidden dynamic structural features and holds the key to the understanding of the different phenomena observed in the glass science, such as deformation, diffusion and glass transition^1^,^11^,^12^,^13^,^14^.

Structurally complicated glasses usually exhibit a complex relaxation spectrum, such as some molecular glasses, which possess a hierarchical structure composed of main molecular chains, chain branches and side groups^15^. Once agitated in a typical relaxation experiment, they tend to display a relaxation spectrum with distinct peaks, such as the α (primary) peak, the β (secondary) peak, even the γ peak and so on and so forth^16^,^17^. By comparison, glassy metals or metallic glasses, the newcomer to the glassy family about 50 years ago, are simply made up of atoms^18^,^19^. Hence, it was once thought that there could be only one α peak on their relaxation spectrum. It was only until recently did people find that metallic glasses could also exhibit secondary β relaxations^20^, which can bring about macroscopic tensile ductility^14^, assist the diffusion of small atoms within an amorphous structure^11^, and trigger the activation of the shear-transformation-zones (STZ)^21^,^22^ or ‘flow units' in metallic glasses^23^. Because of the fundamental importance and the technological relevance, the discovery of the β relaxation in metallic glasses has triggered a surge of research interest recently in the glass field^11^,^14^,^21^,^22^,^24^,^25^. However, here we would like to provide the compelling experimental evidence showing that there are not one but two β relaxations in metallic glasses, which is further against the conventional thinking that metallic glasses can only possess a simple structural relaxation spectrum because of their apparent structural simplicity. More importantly, in light of the connection between the slow β relaxation and STZ activation^21^,^26^, the discovery of the fast β relaxation provides us the important insight into the dynamic origin of inelasticity that triggers the yielding process in metallic glasses.

## Results

### Dynamic mechanical analysis

**Figure 1:**
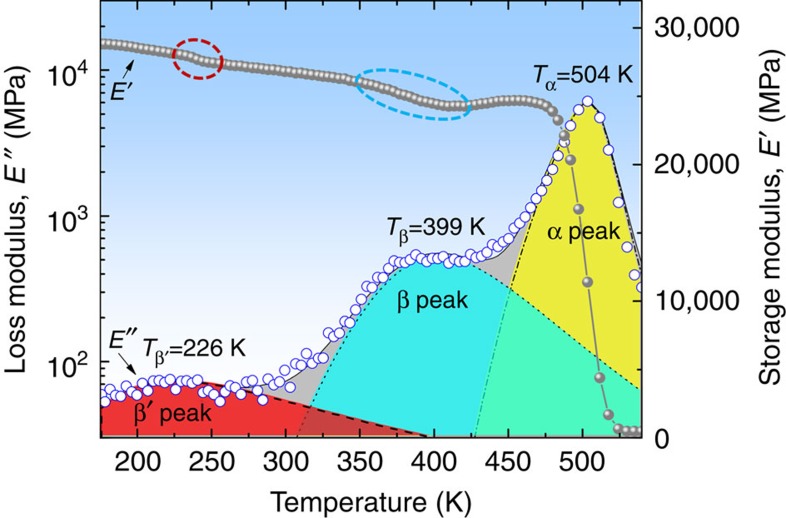
The emergence of two secondary relaxations in the La-based metallic glass on the isochronal dynamical mechanical spectrum. The temperature dependence of loss modulus, *E*″ and storage modulus, *E*′, was obtained at the testing frequency of 1 Hz and heating rate of 3 K min^−1^. The red and green regions represent the Cole–Cole (C–C) fitting of the fast β′ and slow β relaxation, respectively, while the yellow region represents the H–N fitting of the α-relaxation.

For this study, we chose a La-based bulk metallic glass as the model material, which has the chemical composition of La_56.16_Ce_14.04_Ni_19.8_Al_10_ (in atomic %). As seen in the later text and Supplementary Information, this La-based metallic glass has an excellent thermal stability and pronounced β relaxation. The amorphous nature of the La-based alloy is confirmed by means of X-ray diffraction as well as differential scanning calorimetry (DSC; see Supplementary Figs 1 and 2). Subsequently, its relaxation behaviours were characterized using the TA Q800 dynamical mechanical analyzer (DMA) across a wide range of temperatures and frequencies. Figure 1 presents the isochronal DMA results of the alloy, including the loss modulus *E*″ and the storage modulus *E*′, as obtained from the temperature *T*=173–540 K at the single testing frequency *f*=1 Hz. Note that, as compared with the conventional DMA tests, the testing temperature range in our experiments was significantly extended with the starting point lowered down to 173 K rather than the ambient temperature. As seen in Fig. 1, it is striking that the *E*″ curve shows more than two distinct peaks in the given testing temperature range: besides a pronounced β- (or secondary) relaxation peak at 399 K and a α- (or main) relaxation peak at 504 K, there is another relaxation spectrum peaked at a lower temperature of 226 K, which spans a wide temperature range from below 173 to above 300 K, indicative of a broad distribution of relaxation times similar to the β relaxation. However, the amplitude or strength of the newly found peak is rather low, only about 1/100 of the α relaxation, contrasting the well-known strength of a typical β relaxation process, which is usually lower than that of α relaxation by about 1/10 (refs 27, 28). Moreover, it is worthy to mention that the storage modulus *E*′ shows a relatively small but noticeable drop at the temperature corresponding to the low-*T* relaxation peak (the encircled areas in Fig. 1), which is similar to the α and β relaxation processes and further confirms the existence of the newly found relaxation peak. For the sake of discussion, this low-*T* relaxation process is herein termed as the fast β′ relaxation as opposed to the conventional slow β relaxation.

**Figure 2:**
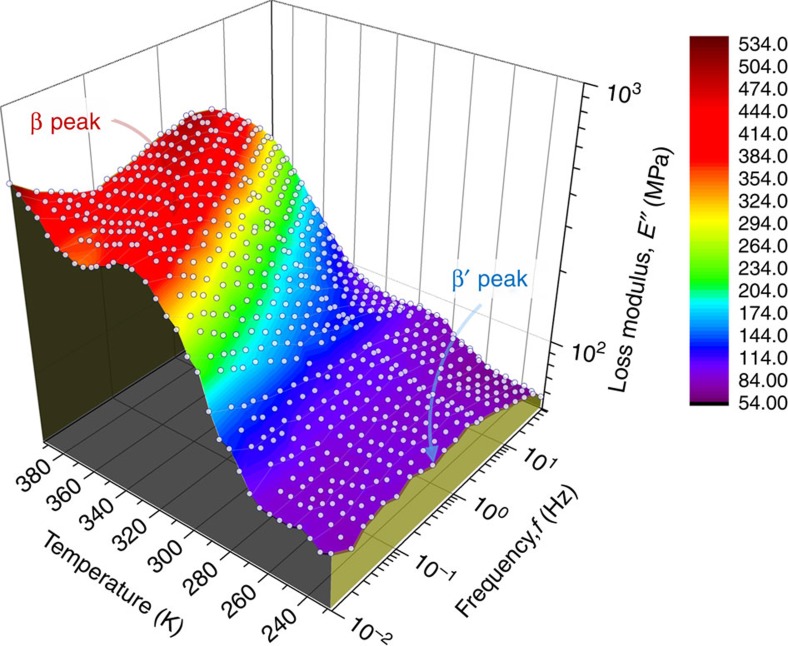
The three-dimensional surface plot of the loss modulus *E*″ as a function of the testing temperature and frequency obtained from extensive DMA tests. Note that the experimental data (white dots) are superimposed on the surface plot.

To obtain a holistic understanding of these secondary relaxation processes, the isothermal mechanical relaxation spectra *E*″(*ω*) of the La-based metallic glass were measured in the frequency window of 1 × 10^−2^≤*f*≤100 Hz by incrementally adjusting the testing temperature from 228 to 393 K at a step of 5 K. Figure 2 displays the three-dimensional surface plot of all the isothermal data so obtained from the DMA tests. Evidently, the mechanical relaxation behaviour of the alloy is dominated by the β′ relaxation at the testing temperature as low as 228 K, which is in agreement with the previous isochronal result shown in Fig. 1. As the testing temperature rises, the signal of the β-relaxation gradually emerges from the low-*f* end and ultimately prevails on the isothermal spectrum; by comparison, the signal of the β′-mode relaxation weakens with the increasing temperature, which merges into that of the β-relaxation in the form of ‘shoulder' and ‘excessive wings' successfully and finally becomes indistinguishable at the high temperature (Supplementary Fig. 3a–f). This behaviour is very similar to the temperature-driven transition of β to α relaxation, as reported in the literature^25^,^28^.

### Thermodynamic characterization of secondary relaxations

To gain quantitative understanding of the above unusual relaxation behaviour, we further analyse the mechanical loss spectrum of the La-based alloy using the general Havriliak–Negami (H–N) method^29^ (see Methods), according to which the complex modulus E* of a glassy solid can be expressed as 
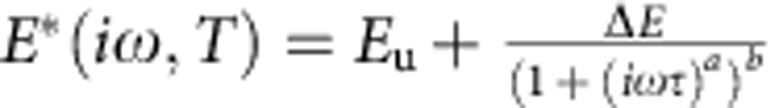
, where *E*_u_ denotes the unrelaxed modulus; *ω* is the angular frequency; *τ* is the relaxation time; and the relaxation strength Δ*E*=*E*_u_−*E*_r_ in which *E*_r_ is the relaxed modulus of the glass while the parameter *a* and *b* respectively describe the broadness and asymmetry of the relaxation spectrum to be analysed. For brevity, the detailed discussion of the physical meanings of *a* and *b* is deferred to the Methods. Note that the above H–N equation is essentially empirical in nature which could be fitted to any type of relaxation spectrum like the well-known Kolrausch–Williams–Watts (KWW) relation^30^; however, the H–N equation yields richer information on the dynamics of a relaxation behaviour as it involves more fitting parameters^31^ and has its origin rooted in the Debye relaxation^32^, the dynamics of which follows the Arrhenius behaviour and corresponds to *a*=*b*=1.

**Figure 3:**
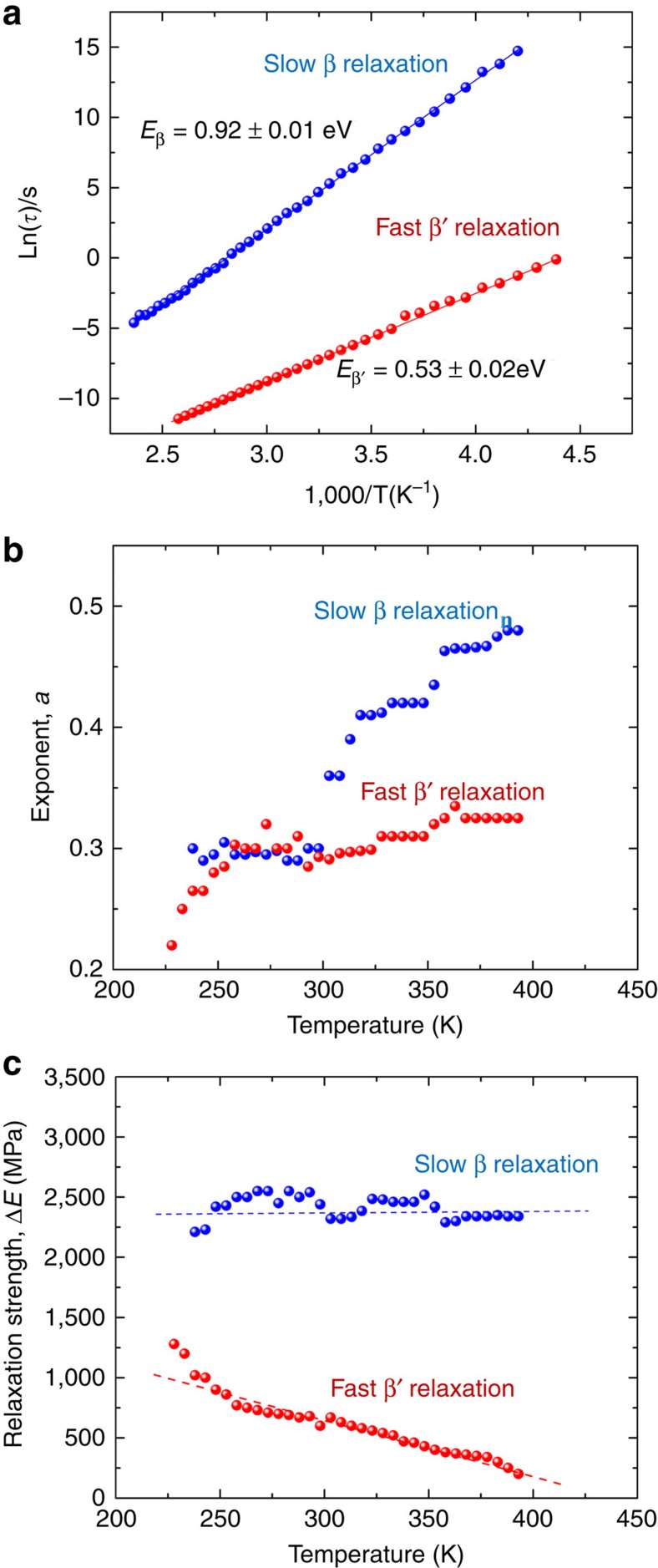
The relaxation properties obtained through the H–N fitting of the experimental data. The temperature dependence of (**a**) the relaxation times, (**b**) the exponent *a*, as the indicator for the broadness of the relaxation spectrum and (**c**) the relaxation strength.

By fitting the relaxation spectra to the H–N relation (Supplementary Note 2), we are able to precisely pinpoint the relaxation times of different relaxation processes and to obtain the corresponding relaxation strengths *ΔE* and exponents (*a* and *b*). Like the slow β relaxation in other types of glasses such as polymers^27^, the H–N fitting of the DMA curves yields the unity *b* exponent for both the fast and slow secondary relaxations in our La-based alloy (Supplementary Table 1), which conforms to the localized Cole–Cole relaxation process^33^ (*b*=1) well known in the relaxation literature^27^. As shown in Fig. 3a, the relaxation times (*τ*_β_ and *τ*_β′_) for the β and β′ secondary relaxations clearly follow the Arrhenius behaviour. By fitting the experimental data to the Arrhenius equation, we can thus obtain the activation energy *E*_β_=0.92 eV for the β relaxation, which obeys the empirical relation *E*_β_=26(±2)*RT*_g_ established for the ordinary β relaxation in a variety of glassy systems^21^,^34^, where *R* is the gas constant and *T*_g_ the glass transition temperature. By comparison, the activation energy *E*_β′_ of the β′ relaxation is rather low, which is ∼0.53 eV and roughly one half of that of the β relaxation (Fig. 3a).

In addition to the mean of the relaxation times, the H–N fitting also yields the stretching exponent *a* for both secondary relaxations at different temperatures. From the theoretical viewpoint, the stretching exponent *a* measures the width of the distribution of the relaxation times: the smaller is *a* the wider is the relaxation time distribution and vice versa. Note that the stretching exponent can be also taken as an indicator of the dynamic heterogeneity in amorphous materials according to the literature^35^. As shown in Fig. 3b, the value of the *a* exponent for both secondary relaxations increases with the temperature; however, the *a* exponent for the fast β′ relaxation rises at a slower rate and is lower than that of the slow β relaxation at the same testing temperature. This behaviour indicates that during the whole structural evolution process, the fast β′ relaxation entails a wider relaxation time distribution than the slow β relaxation. Moreover, it is interesting to point out that the *a* exponent for the slow β relaxation remains constant below 300 K and subsequently increases with temperature. This trend is quite similar to the temperature-dependent behaviour of the stretching parameter, *β*_KWW_, as derived through the KWW fitting of the stress relaxation curves obtained from a similar La-based metallic glass^36^.

Furthermore, we can also compare the relaxation strengths of both secondary relaxation processes obtained through the H–N fitting, which can be regarded as the measure of the total dissipated mechanical energy through the individual relaxation processes during DMA testing. As shown in Fig. 3c, the relaxation strength of the slow β relaxation remains nearly a constant over the temperature from ∼240 to ∼400 K, conforming to the typical feature exhibited by the Johari–Goldstein (JG) relaxations^28^. On the contrary, the relaxation strength of the fast β′ mode plunges as the temperature rises. These interesting trends indicate that the contribution of the fast β′ relaxation to the total energy dissipation is diminishing at the high temperatures while that of the slow β relaxation keep almost invariant within the temperature range we studied.

## Discussion

On the basis of the above analyses, it is clear that, like the slow β relaxation, the fast β′ relaxation is also a localized relaxation process whose relaxation times follow the Arrhenius relation, as shown in Fig. 3a. However, unlike the slow β relaxation, the fast β′ relaxation diminishes in strength with the increasing temperature; in addition, the fast β′ relaxation possesses a rather low activation energy (∼0.5 eV), only about one half of the slow β relaxation. These important relaxation characteristics provide us clues to understand the possible physical/chemical origins of these secondary relaxations. First, let us discuss the possible chemical origin of the fast β′ relaxation. As discovered by Yu *et al.*^24^, pronounced secondary (slow) β relaxation can be easily observed in the metallic glass systems that have all constituent atomic pairs with similar negative values of mixing enthalpy. Recently, through a comprehensive and systematic study, Zhu *et al.*^37^ further pointed out that the prominent β peak in metallic glasses can be attributed to the presence of highly ‘mobile' atomic pairs, such as the La–Ni pair in La-based metallic glasses, which lowers down the onset temperature for β relaxation such that a prominent β peak can be separated out from the α peak. Since the chemical composition of our metallic glass (La_56.16_Ce_14.04_Ni_19.8_Al_10_) contains both La and Ce while La–Ni–Al and Ce–Ni–Al are known to be the prototypical compositions showing pronounced β peaks^24^,^28^, it is likely that there might be two types of mobile atomic pairs, with one being La–Ni^37^ and the other possibly being associated with Ce, to trigger the secondary relaxations one after another in our metallic glass. If that was the case, it can be inferred that there should be a large difference between the onset temperatures for β relaxations in La- and Ce-based metallic glasses; otherwise the two secondary relaxation peaks cannot distinguish themselves from each other. Indeed, the recent experiments conducted by Yu *et al.*^38^ showed that the β relaxation behaviour and its onset temperature in the (La, Ce)-based metallic glass can be systematically changed with the substitution of La with Ce, which supports the idea that there could be two types of atomic pairs to trigger the succession of β′ and β relaxations, as seen in our experiments. However, further research is still needed to understand the details of the chemical effect.

**Figure 4:**
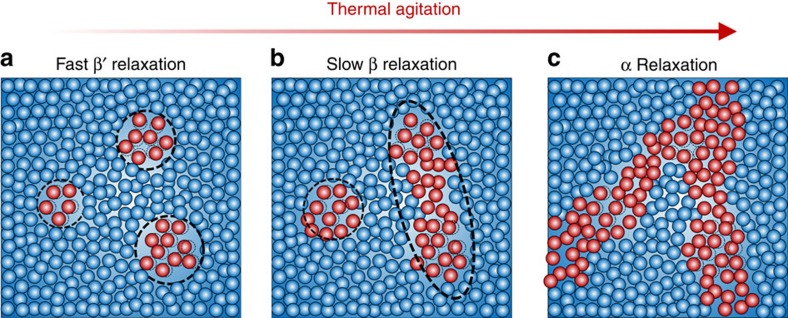
The illustrated mechanisms of mechanical relaxations in metallic glass. (**a**) The fast β′ relaxation is associated with local structural rearrangements, (**b**) the slow β relaxation with the linkage of local structural rearrangements, and (**c**) the α relaxation with the percolation of the local relaxations through the glass ‘matrix'. Note that the red and blue spheres represent mobile and less mobile atoms respectively.

Next we would like to discuss the possible physical origin of the fast β′ relaxation in comparison with that of the slow β relaxation. As pointed out by Yu *et al.*^11^, the slow β relaxation comes about as a result of the excitations of many β-events through ‘string-like' atomic configurations. Physically, this is similar to a typical local STZ event that involves a cascade of ‘cage-breaking' events. On the fundamental level, this explains why the slow β relaxation and STZ activation share almost the same activation energy as discovered in ref. 21. Here, each ‘cage-breaking' event is defined as the activation of the individual group of loosely bonded or ‘liquid-like' atoms caged within their elastic surroundings, as shown in Fig. 4a. According to many theories and models in the literature, such as the random first order transition theory^39^,^40^, the atomic stress theory^41^, the two-order-parameter model^8^, the flow unit model^23^,^42^ or the interstitial model^43^, these loosely bonded atoms may be viewed as ‘defects' that can trigger the cascade of cage-breaking events and ultimately leads to plastic flow in metallic glasses. According to the previous works^44^,^45^,^46^, the activation energy Δ*E*_id_ for the individual ‘defect' appears smaller than that (∼26 *RT*_g_) for a typical STZ event or slow β relaxation. Furthermore, the activation energy Δ*E*_id_ was already measured for a variety of metallic glasses through anelasticity with the measured value of Δ*E*_id_ ranging from 0.3 to 0.5 eV (ref. 44), which compares very well with the activation energies derived from atomistic simulations for the individual activation process, such as ∼0.3 eV in the Ni–Zr (ref. 45) and ∼0.4 eV in the Cu–Ti (ref. 46). Following the nanoindentation approach as detailed in ref. 44, we can also measure the activation energy *ΔE*_id_ corresponding to the anelastic deformation in our metallic glass sample (Supplementary Fig. 4 and Supplementary Note 3). Interestingly, the nanoindentation results yield *ΔE*_id_=0.5 eV (Supplementary Table 2), which is almost identical to that of the fast β′ relaxation. This similarity suggests that, compared with the slow β relaxation, the fast β′ relaxation is physically more confined, which is related to the excitation of an individual local event more than to a cascade of local excitations. This is also consistent with the chemical origin of the β′ relaxation we previously discussed. As the secondary relaxation spreads out from the most mobile to the less mobile atomic pairs, relaxation events should extend from the very local sites to their surroundings.

To summarize, two secondary relaxation processes are identified in the La_56.16_Ce_14.04_Ni_19.8_Al_10_ metallic glass in the current study rather than one secondary relaxation as reported before. As the chemical composition of our metallic glass contains a variety of mobile atomic pairs due to the combination of the very mobile rare-earth elements (that is, La and Ce) with Ni or Al, multiple local relaxation processes arise because of the distinction in the different onset temperatures for secondary relaxations. As a result, this facilitates the detection of the very subtle and much localized relaxation process in the metallic glass with an activation energy of ∼0.5 eV, being as low as that for the individual thermal activation process. On the basis of our findings, a physical picture is emerging which describes how mechanical relaxation is initiated and progresses in metallic glasses. At a low temperature, relaxations are initiated and centred in the very local sites where the most mobile atoms reside (Fig. 4a). As the temperature increases, the relaxations spread out to their surroundings and include less mobile atoms; however, during this stage, these relaxations are still local and confined by the overall elastic ‘matrix' of glass (Fig. 4b). When the temperature becomes sufficiently high, the local relaxation sites finally percolate through the elastic ‘matrix' of glass, resulting in the α relaxation as shown in Fig. 4c.

## Methods

### Casting of the Ce–La–Al–Ni bulk metallic glass

The La–Ce–Ni–Al alloy ingots were prepared by arc-melting mixtures of pure metals in Ti-gettered argon atmosphere. Each ingot was melted at least three times to obtain chemical homogeneity and eventually cast into a water-cooled copper mold to obtain a rectangular plot with the dimension of ∼80 mm × 10 mm × 1 mm. The amorphous structure of the as-cast samples were examined with X-ray diffractrion using Cu-Kα radiation and DSC under a flow of purified argon in a Perkin Elmer Diamond DSC (Supplementary Note 1).

### Dynamic mechanical analysis tests

The relaxation behaviours of the La-based bulk metallic glass were characterized by a TA Q800 DMA, which is a sensitive tool detecting mechanical relaxations in glassy solids as well as supercooled liquids^28^. The dynamic modulus E*(*ω*)=*E*′(*ω*)+*iE*″(*ω*), where the real part *E*′and imaginary part *E*″ represent the storage and loss modulus, respectively, was measured as a function of either the temperature, in the range from 300 to 500 K at a constant heating rate of 0.3 K min^−1^, or the frequency, in the range from 0.01 to 100 Hz at different isothermal temperatures. In the DMA tests, the rectangular specimens (30 mm × 2 mm × 1 mm) were gripped to an oscillating system and subject to sinusoidal single cantilever-bending strains with the limited amplitude of about 0.1%.

### H–N fitting of frequency spectrum

Following the well-establish method for studying the dielectric or mechanical relaxation of polymers^29^, the isothermal frequency-sweep DMA spectra, obtained from the La-based BMG, were analysed using the H–N relaxation model^29^. The H–N relaxation model is the empirical generalization of the Debye relaxation model, which accounts for both the asymmetry and broadness of the relaxation curve by adding two exponential parameters to the well-known Debye equation. For DMA tests, this gives 
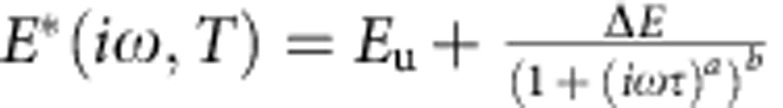
, where E* and *E*_u_ are the dynamic and unrelaxed modulus, respectively, with the relaxation strength Δ*E*=*E*_r_−*E*_u_; *E*_r_ is the relaxed modulus and *τ* is the characteristic relaxation time. In general, the exponents *a* and *b* respectively describe the broadness and asymmetry of the corresponding relaxation process, obeying the conditions 0<*a*≤1 and 0<*ȧb*≤1. It is worthwhile to note that the H–N equation reduces to the Debye equation for *a*=*b*=1, and the Cole–Cole equation for *b*=1and 0<*a*≤1 (ref. 33), and the Cole –Davidson equation for *a*=1 and 0<*b*≤1 (ref. 47). According to Alvarez *et al.*^31^, there exist a relationship between the frequency domain H–N relation and the KWW relaxation functions often used in time domains and a specific KWW exponent corresponds a specific H–N (*a*,*b*) pair. Nevertheless, the KWW function cannot be considered as an ‘universal', since it fails to find a KWW exponent for all *a*,*b* values^31^. Note that the main structural process and secondary relaxation in polymers have been well described by a H–N and Cole–Cole equation, respectively^27^.

### Differential scanning calorimetry

DSC was applied to examine the kinetics of glass transition in the La-based BMG and a set of DSC curves were obtained at different heating rates, ranging from 5 to 80 K min^−1^ (Supplementary Fig. 2 and Supplementary Note 1).

## Additional information

**How to cite this article:** Wang, Q. *et al.* Unusual fast secondary relaxation in metallic glass. *Nat. Commun.* 6:7876 doi: 10.1038/ncomms8876 (2015).

## Supplementary Material

Supplementary Information Supplementary Figures 1-4, Supplementary Tables 1-2, Supplementary Notes 1-3 and Supplementary References
