# Supplementary material for: Unusual fast secondary relaxation in metallic glass
Source: Nat Commun. 2015 Jul 24;6:7876. doi: 10.1038/ncomms8876 (PMC4525198; doi:10.1038/ncomms8876)
Supplement: Supplementary Information — Supplementary Figures 1-4, Supplementary Tables 1-2, Supplementary Notes 1-3 and Supplementary References [file ncomms8876-s1.pdf]

Supplementary Figures

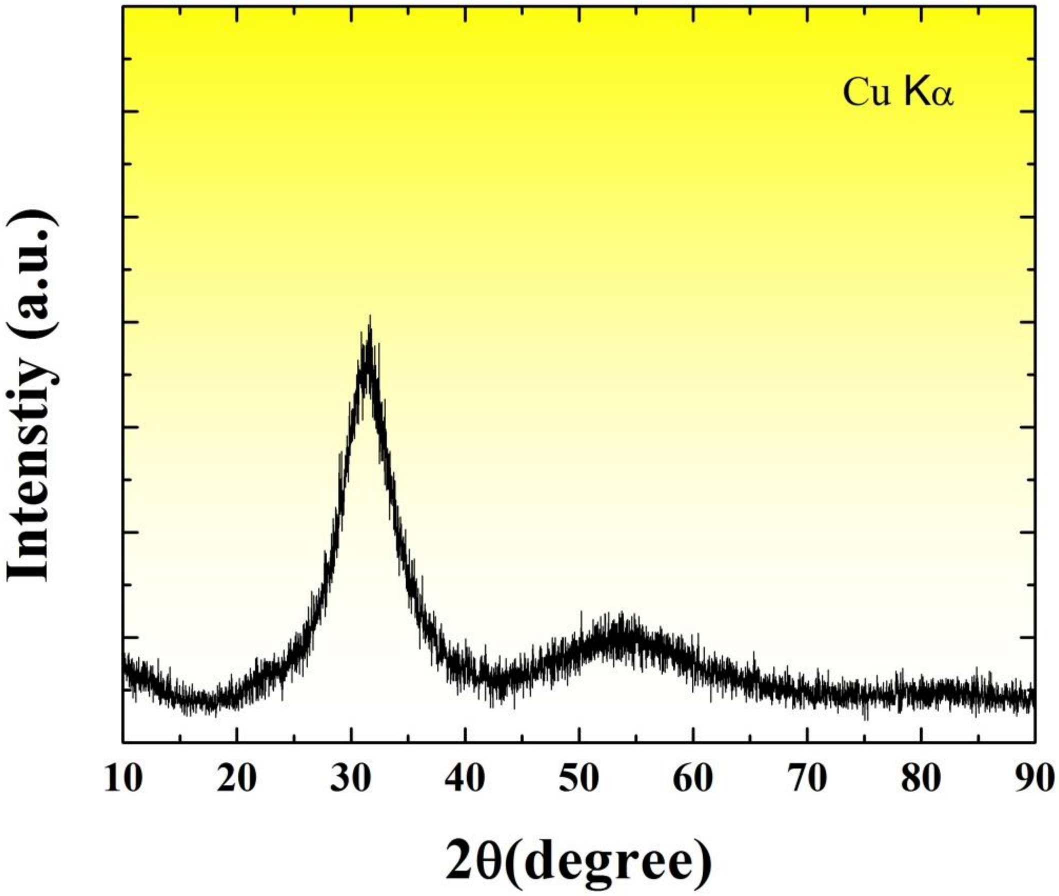

Supplementary Figure 1. X-ray diffraction pattern of the as-cast

$La_{56.16}Ce_{14.04}Ni_{19.8}Al_{10}$ BMG alloy

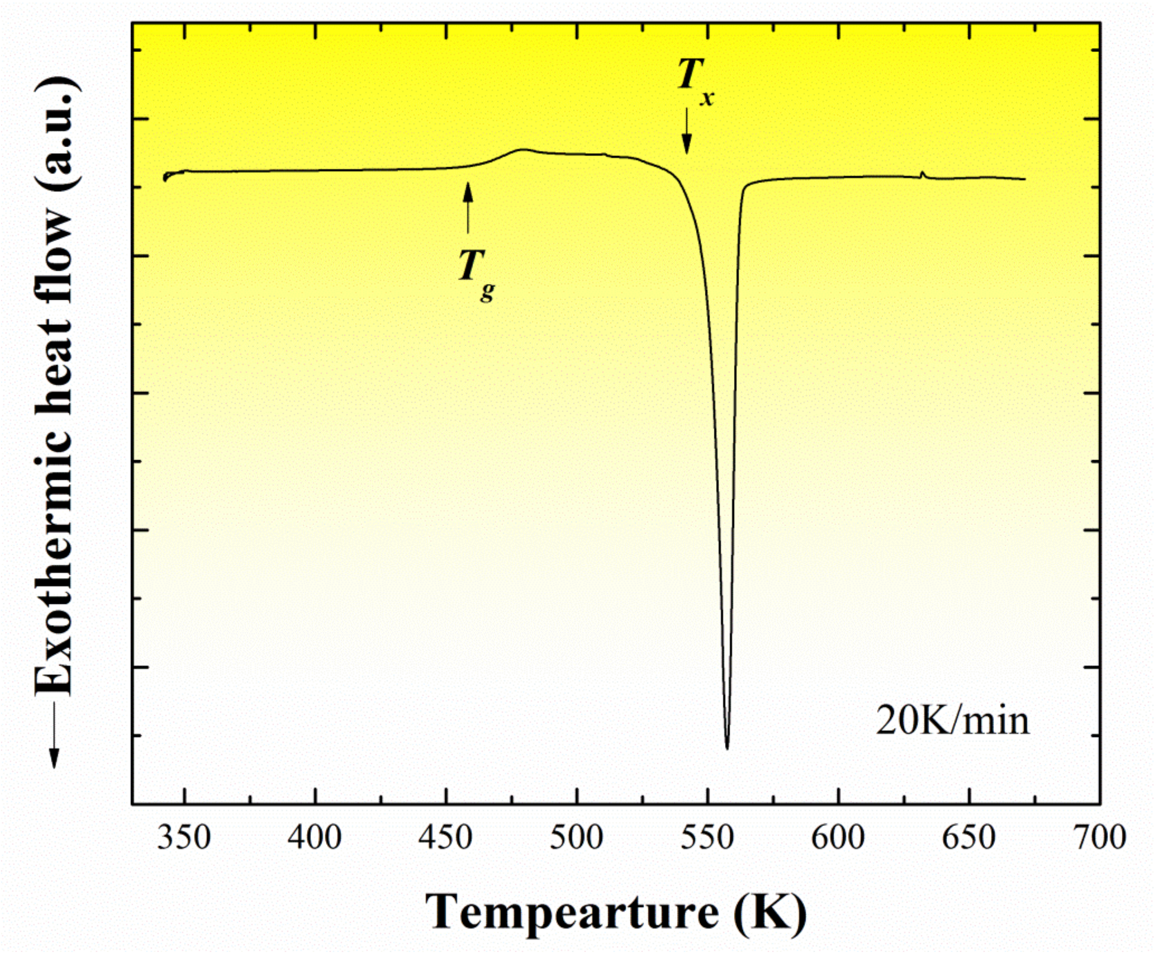

Supplementary Figure 2. The DSC curve of the as-cast $La_{56.16}Ce_{14.04}Ni_{19.8}Al_{10}$ BMG alloy obtained at 20K/min

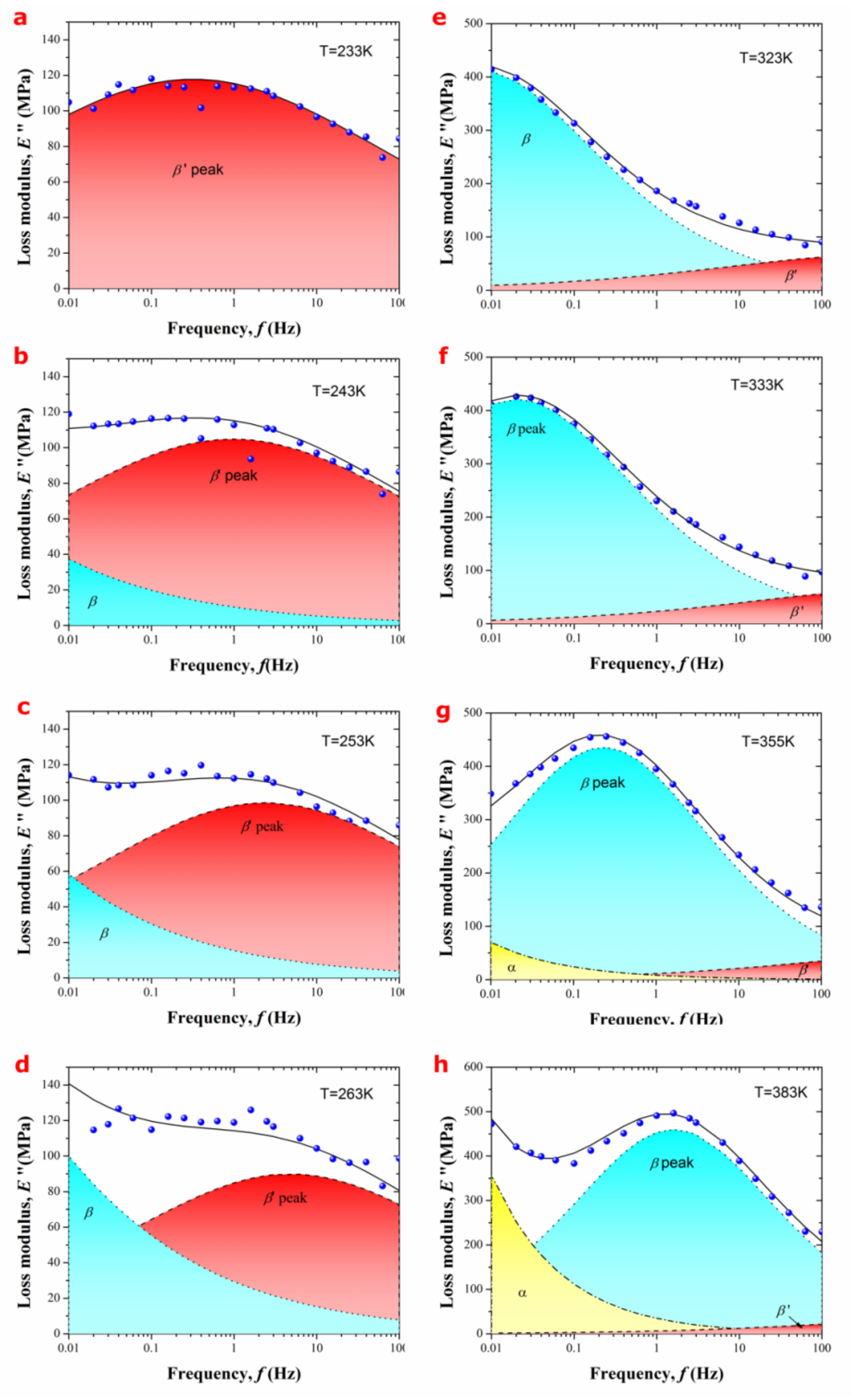

Supplementary Figure 3. Frequency-dependent mechanical loss spectra obtained at various temperatures (only a selection of temperature is shown for readability). The solid lines are fits with a Cole-Cole (C-C) function for lower temperatures, e. g. 233K (a), with the sum of two C-C functions for T⩾238K, a Cole-Davidson (C-D) and two C-C functions for T⩾355K, respectively. The dashed, dotted curves at the bottom show the C-C curves for fast β'- and slow β- relaxations, respectively, and the dash-dotted one for C-D curves α-relaxation.

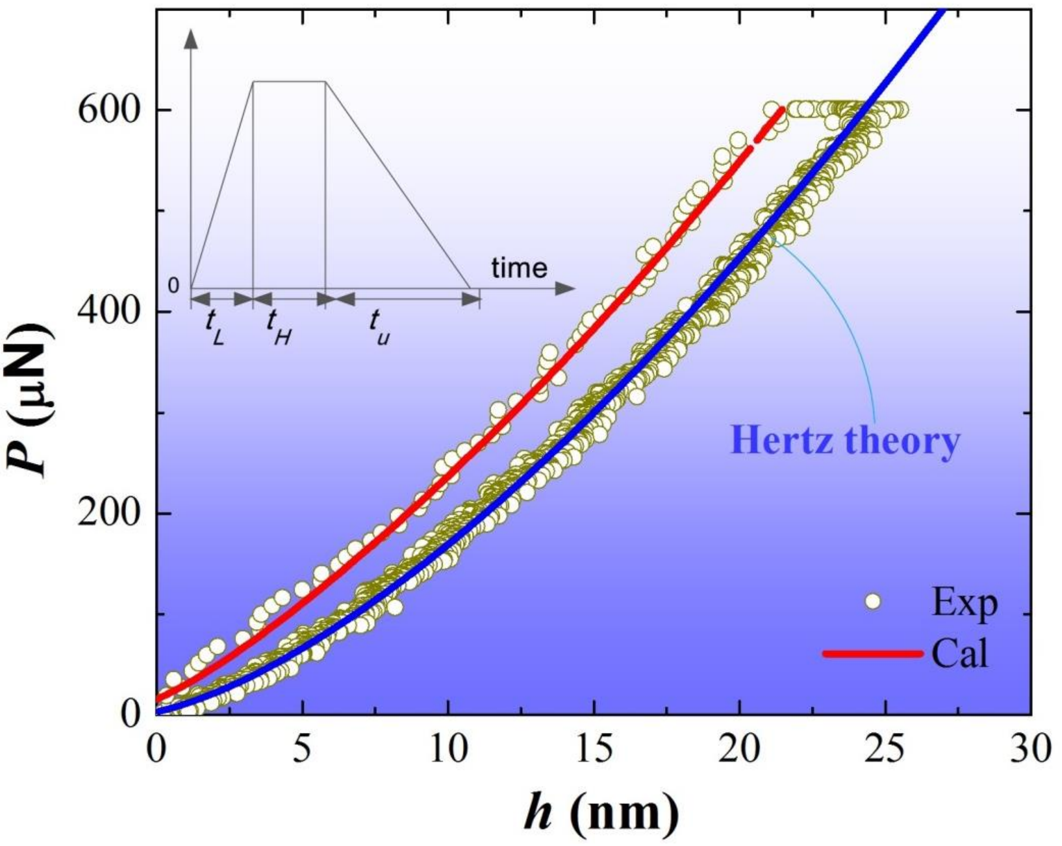

Supplementary Figure 4. The typical indentation load-displacement curve revealing the anelasticity in the metallic glass (the inset: the schematic of the indentation load function)

Supplementary Tables

Supplementary Table 1. The fitting parameters including H-N function exponents (*a*, *b*), characteristic relaxation time (τ) and relaxation strength (ΔE) for the loss modulus spectra of the La-Ce-Ni-Al BMG

	Fast β' secondary relaxation				Slow β secondary relaxation				α-relaxation			
T/K	τ/s	*a*	*b*	ΔE/MPa	τ(s)	*a*	*b*	ΔE/MPa	τ/s	a	b	ΔE/MPa
228	0.900	0.220	1	1280	-	-	-	-	-	-	-	-
233	0.499	0.250	1	1200	-	-	-	-	-	-	-	-
238	0.283	0.265	1	1020	2.49×10^6	0.300	1	2210	-	-	-	
243	0.165	0.263	1	1000	9.8×10^6	0.290	1	2230	-	-	-	-
248	0.11	0.280	1	900	5.65×10^6	0.300	1	2420	-	-	-	-
253	0.066	0.287	1	860	1.85×10^5	0.320	1	2430	-	-	-	-
258	0.0468	0.300	1	770	8.33×10^4	0.300	1	2500	-	-	-	-

263	0.033	0.300	1	750	3.30×10^{4}	0.295	1	2550	-	-	-	-
268	0.0209	0.305	1	760	1.57×10^{4}	0.300	1	2500	-	-	-	-
273	0.0127	0.300	1	740	8.13×10^{3}	0.290	1	2550	-	-	-	-
278	0.0165	0.300	1	730	4.45×10^{3}	0.298	1	2450	-	-	-	-
283	0.0081	0.300	1	720	2.48×10^{3}	0.290	1	2500	-	-	-	-
288	0.00293	0.310	1	670	1.09×10^{3}	0.290	1	2500	-	-	-	-
293	0.00202	0.320	1	680	6.06×10^{2}	0.300	1	2540	-	-	-	-
298	0.00141	0.295	1	610	4.15×10^{2}	0.295	1	2420	-	-	-	-
303	9.97×10^{-4}	0.293	1	670	198.02	0.360	1	2320	-	-	-	-
308	7.21×10^{-4}	0.291	1	630	108.11	0.360	1	2320	-	-	-	-
313	5.15×10^{-4}	0.296	1	600	57.08	0.390	1	2335	-	-	-	-

318	3.76×10^{-4}	0.295	1	580	35.69	0.410	1	2385	-	-	-	-
323	2.77×10^{-4}	0.298	1	560	24.38	0.410	1	2485	-	-	-	-
328	2.06×10^{-4}	0.299	1	543	14.08	0.412	1	2480	-	-	-	-
333	1.55×10^{-4}	0.310	1	520	8.13	0.430	1	2460	-	-	-	-
338	1.17×10^{-4}	0.310	1	470	4.93	0.420	1	2460	-	-	-	-
343	8.93×10^{-5}	0.310	1	460	3.10	0.420	1	2460	-	-	-	-
348	6.87×10^{-5}	0.310	1	430	2.07	0.420	1	2520	-	-	-	-
353	5.32×10^{-5}	0.320	1	400	1.35	0.435	1	2420	8.86×10^{6}	1	0.465	22500
358	4.15×10^{-5}	0.325	1	380	0.69	0.463	1	2290	1.66×10^{6}	1	0.465	22500
363	3.26×10^{-5}	0.335	1	370	0.48	0.465	1	2300	6.80×10^{5}	1	0.454	22500
368	2.58×10^{-5}	0.325	1	360	0.36	0.465	1	2340	2.81×10^{5}	1	0.488	22500

373	2.05×10^{-5}	0.325	1	350	0.23	0.454	1	2400	1.18×10^{5}	1	0.490	22500
378	1.64×10^{-5}	0.325	1	340	0.17	0.467	1	2340	6.70×10^{4}	1	0.490	22500
383	1.32×10^{-5}	0.325	1	300	0.10	0.475	1	2350	2.65×10^{4}	1	0.505	21200
388	1.07×10^{-5}	0.325	1	250	0.07	0.48	1	2340	1.70×10^{4}	1	0.504	20000
393	8.73×10^{-6}	0.325	1	200	0.06	0.47	1	2340	1.40×10^{4}	1	0.506	19000

Supplementary Table 2. The rheological properties obtained from anelasticity of the La-based metallic glass at the ambient temperature.

Indentation Load (μN)	μ (GPa)	G_I (GPa)	t_c (ms)	ΔG (eV)
400	24.73±0.52	17.67±0.65	2.61±1	0.55±0.02
500	24.98±0.09	16.39±0.38	1.41±1	0.54±0.01
600	25.63±0.39	17.07±0.40	2.06±1	0.55±0.02

Supplementary Notes

Supplementary Note 1

As shown in Supplementary Figure 1, the XRD pattern of an as-cast $La_{56.16}Ce_{14.04}Ni_{19.8}Al_{10}$ consists of only a broad diffraction maximum without any detectable sharp Bragg peaks from crystalline phases. Therefore, it is considered that the amorphous structure of the as-cast alloys is verified within the XRD resolution limitation. Supplementary Figure 2 shows a typical DSC trace obtained from the as-cast La based BMG alloy at the constant heating rate of 20K/min. The DSC curve exhibits a distinct endothermic reaction corresponding to the glass transition, followed by a wide supercooled liquid region before a crystalline exothermic peak shown at a higher temperature during the continuous heating. The glass transition temperature, T_g, the onset crystallization temperature, T_x and the supercooled liquid region defined as the temperature interval $\Delta T_x = T_x - T_g$ are determined to 461, 549 and 88K, respectively. Those observations further confirm the amorphousness of the as-cast alloy and indicate that the La-based BMG alloy has a high thermal stability with respect to crystallization, which enables the measurement of dynamical mechanical properties over a sufficiently wide temperature and/or time window.

Supplementary Note 2

In the present work, we concentrate on the dynamic mechanical spectrum of the La-based BMG over a relatively large temperature window (from 233K to 393K) with applied frequency varying from 0.01 to100Hz for a thorough investigation of the secondary relaxations. As shown in Supplementary Figure 3, the isothermal mechanical loss spectra of the La-Ce BMG alloy can be fitted very well to the Hvariliak Negami (H-N) function[1-5] $E^*(i\omega,T) = E_u + \frac{\Delta E}{(1+(i\omega\tau)^a)^b}$, where E^* and E_u denote respectively the dynamic and un-relaxed modulus, the relaxation strength $\Delta E = E_r - E_u$ with E_r being the relaxed modulus, and τ is the characteristic relaxation time. Note that the parameter a and b describe the asymmetry and broadness of the corresponding relaxation spectra, respectively, and the values of the fitting parameters are listed in Supplementary Table 1.

From the typical loss modulus spectra, $E''(\omega)$, a pronounced mechanical loss peak can be observed at lower temperatures, e. g. 228 or 233K [Supplementary Figure 3(a)], before the ordinary secondary (slow β) relaxation sets in and gradually becomes dominant with the increasing testing temperature[Supplementary Figure 3 (b)-(h)]. The mechanical loss spectra of the La based BMG are successively dominated by the two different sub-T_g secondary relaxations shifting through the given frequency window. As pointed out in the main text, the identified low-T loss peak corresponds to another faster secondary (β') relaxation process preceding the slow β relaxation in time. Here it should be noted that an increase of the loss modulus towards the lowest frequencies is visible at the temperatures above 355K,

which can be attributed to the α relaxation whose peak lies outside the experimental frequency window [Supplementary Figure 3(g) and (h)]. Furthermore, as shown in Supplementary Figure 3, the H-N fitting allows one to reproduce the main spectral features of the investigated La-based metallic glasses assuming simple superposition of the fast and slow secondary relaxations with the α relaxation. It is worth noting that not only the slow β but also the fast β' relaxation was fitted to the Cole-Cole (C-C) equation (b=1) [6], while the main α relaxation process to the Cole-Davidson (C-D) relation (a=1)[7](see **Methods** in the main text). For instance, at the low temperatures, i.e. 173 or 178K, the loss spectra corresponding to the fast β' relaxation process can be well fitted by using just only one the C-C curve. As the temperature is increased, an additional C-C equation is required to well fit the mechanical loss spectrum which corresponds to the slow β process over the investigated frequency range. At the temperatures above 355K, in addition to the two sub-T_g secondary relaxations, the primary (α) relaxation process is found and merges with the low-frequency tail of the slow β-relaxation spectrum [Supplementary Figure 3 (f)-(h)]. As a result, one additional C-D equation is required to fit the mechanical loss spectra over the investigated frequency range.

Supplementary Note 3

Recently, there were substantial studies which revealed that the slow β relaxation in metallic glasses can be linked to a typical "shear transformation" (ST) event or the activation of a hidden "flow unit" because of the nearly same activation energy on the

order of ~ 1 eV[8-11]. As such, a legitimate question to ask is: given the presence of the fast β' relaxation, what kind of deformation process can it be possibly linked with? Since a ST event signals the onset of local yielding and the activation energy of the fast β' relaxation is less than that of the slow β relaxation, it is sensible to hypothesize that the fast β' relaxation process might be linked to the inelastic deformation process in metallic glasses prior to yielding, namely, anelasticity. To characterize the activation energy for anelasticity in our La-based alloy, we carried out spherical nanoindentation tests following the method detailed in the previous work[12]. The basic idea is to apply a small indentation load at a fast rate and subsequently do the unloading slowly after load hold for some time, as illustrated by the inset of Supplementary Figure 4. In this way, both anelastic and elastic strain can be revealed and characterized in the same test[12].

Supplementary Figure 4 presents one typical load (P) versus displacement (h) curve obtained from indenting the La-based metallic glass at the peak load of 600μN and the loading time t_L of 0.005s. It is evident that the P-h curve returns to the zero displacement after the full unloading, indicative of the elastic nature of the material's deformation. However, due to the short loading time and thus ultrafast strain rate, the loading curve deviates from the Hertzian theory which conforms to the character of anelasticity. According to the mean-field model[12], the loading curve of the spherical indentation can be fitted to the following rheological relation:

$$h(t)^{3/2} = \frac{3P(t)(1-\upsilon)}{8\sqrt{R}G_I} - \frac{3\dot{P}(1-\upsilon)(\mu - G_I)t_c}{8\sqrt{R}G_I\mu}\left[1-\exp\left(-\frac{t}{t_c}\right)\right] \quad \text{(S1)}$$

where G_I denotes the quasi-static (relaxed) shear modulus, μ the unrelaxed shear modulus, R the indenter tip radius (=5 μm), υ the Poisson's ratio, $\dot{P}$ the loading rate and t_c the apparent relaxation time $t_c = \exp[\Delta G/(kT)]/(2\omega)$, in which ΔG is the activation energy against the thermal activation processes occurring during anelasticity and ω (~10^{13}) is approximated as the Debye frequency. Note that Eq.(S1) is degenerated to the Hertzian theory for $t >> t_c$. By fitting Eq. (S1) to both the loading and unloading curves (Supplementary Figure 4), we can easily obtain G_I, μ and t_c for T = 299K (the ambient temperature). Thus, the activation energy ΔG against the local configurational transition during anelasticity can be also estimated, as tabulated in Supplementary Table 2. Very interestingly, the obtained activation energy (~0.5 eV) for the anelastic deformation in the La-based metallic glass is very close to that for the fast β'-relaxation but significantly lower than that (~0.92 eV) for the slow β relaxation. In addition, the relaxation time (~2 ms) obtained from anelasticity is also close to that of the fast β' relaxation around the similar temperature. Therefore, these important findings are supportive of our previous hypothesis that anelasticity can be correlated with the fast β' relaxation in the La-based metallic glass, in analogous to the local yielding being correlated with the slow β relaxation[9].

Supplementary References

1 Havriliak, S. & Negami, S. A complex plane representation of dielectric and

mechanical relaxation processes in some polymers. *Polymer* 8, 161-210, doi:10.1016/0032-3861(67)90021-3 (1967).

2 Havriliak Jr., S. & Havriliak, S. J. Comparison of the Havriliak-Negami and stretched exponential functions. *Polymer* 37, 4107-4110 (1996).

3 Havriliak Jr., S. & Havriliak, S. J. Results from an unbiased analysis of nearly 1000 sets of relaxation data. *J. Non-Cryst. Solids* 172-174, 297-310 (1994).

4 Cui, J. *et al.* Rheological behavior of Cu–Zr-based metallic glass in the supercooled liquid region. *Journal of Alloys and Compounds* 592, 189-195, doi:10.1016/j.jallcom.2014.01.014 (2014).

5 Alvarez, F., Alegra, A. & Colmenero, J. Relationship between the time-domain Kohlrausch-Williams-Watts and frequency-domain Havriliak-Negami relaxation functions. *Physical Review B* 44, 7306-7312, doi:10.1103/PhysRevB.44.7306 (1991).

6 Cole, K. S. & Cole, R. H. Dispersion and absorption in dielectrics. I. Alternating current characteristics. *J. Chem. Phys.* 9, 341-351 (1941).

7 Davidson, D. W. & Cole, R. H. Dielectric Relaxation in Glycerine *J. Chem. Phys.* 18, 1417 (1950).

8 Yu, H. B., Samwer, K., Wu, Y. & Wang, W. H. Correlation between β Relaxation and Self-Diffusion of the Smallest Constituting Atoms in Metallic Glasses. *Physical Review Letters* 109, doi:10.1103/PhysRevLett.109.095508 (2012).

9 Yu, H. B., Wang, W. H., Bai, H. Y. & Chen, M. W. Relating activation of shear transformation zones to β relaxations in metallic glasses. *Physical Review B* 81, doi:10.1103/PhysRevB.81.220201 (2010).

10 Yu, H. B., Wang, W. H., Bai, H. Y. & Samwer, K. The β -relaxation in metallic glasses. *National Science Review*, doi:10.1093/nsr/nwu018 (2014).

11 Yu, H.-B., Wang, W.-H. & Samwer, K. The β relaxation in metallic glasses: an overview. *materials Today* 16, 183-191, doi:10.1016/j.mattod.2013.05.002 (2013).

12 Huo, L. S., Zeng, J. F., Wang, W. H., Liu, C. T. & Yang, Y. The dependence of shear modulus on dynamic relaxation and evolution of local structural heterogeneity in a metallic glass. *Acta Mater.* 61, 4329-4338 (2013).
